# The Multiple Sclerosis Severity Allele rs10191329^A^ and Cognitive Function: A UK Biobank Study

**DOI:** 10.1002/acn3.70458

**Published:** 2026-06-22

**Authors:** Ioanna Zimianiti, Sheena Waters, Adil Harroud, Pernilla Stridh, Ruth Dobson, Benjamin M. Jacobs

**Affiliations:** ^1^ Centre for Preventive Neurology, Wolfson Institute of Population Health Queen Mary University of London UK; ^2^ Department of Neurology, Royal London Hospital Barts Health NHS Trust UK; ^3^ The Neuro (Montreal Neurological Institute‐Hospital) Montréal Québec Canada; ^4^ Department of Neurology and Neurosurgery McGill University Montréal Québec Canada; ^5^ Department of Human Genetics McGill University Montréal Québec Canada; ^6^ Department of Clinical Neuroscience Karolinska Institute Sweden

**Keywords:** cognitive function, genetics, multiple sclerosis

## Abstract

The genome‐wide association study of Multiple Sclerosis severity linked the genetic variant rs10191329^A^ to long‐term disability and implicated brain resilience as a determinant of outcome. We hypothesised that rs10191329^A^ might influence cognition in other neurological diseases and healthy controls. We explored the relationship between rs10191329^A^ and cognition using reaction time, fluid intelligence, and prospective memory tests in the UK Biobank. We observed weak but directionally consistent associations between rs10191329^A^ and poorer cognition in controls, with similar but non‐significant trends in Multiple Sclerosis, Parkinson's disease, and dementia. Our results support the hypothesis that rs10191329^A^ might affect multiple sclerosis outcomes by affecting brain health.

## 1 Introduction

Multiple Sclerosis (MS) is an autoimmune and degenerative disease of the Central Nervous System (CNS). Preventing long‐term disability remains an unmet need despite effective suppression of inflammatory relapses by existing disease‐modifying treatments [1]. Recently, a genome‐wide association study (GWAS) of > 12,000 MS cases linked an intergenic variant at the *DYSF*‐*ZNF638* locus, rs10191329^A^, to cross‐sectional physical disability [2]. Cell‐type heritability enrichment implicated CNS‐resident cells, and Mendelian Randomisation suggested genetic overlap with educational attainment [2]. Further studies linked rs10191329^A^ to brain atrophy, elevated serum Neurofilament light chain (NFL) levels and retinal layer atrophy, markers of neuroaxonal degeneration [3, 4, 5]. Replication attempts in smaller cohorts have demonstrated consistent effect directions [6, 7, 8]. These findings reinforce a CNS‐mediated mechanism of MS progression. The concept that neuronal resilience modulates disease progression has support across neurological disorders, including MS, Alzheimer's disease (AD) and Parkinson's Disease (PD) [9, 10, 11, 12].

We therefore hypothesised that rs10191329^A^ might influence cognition in MS, healthy controls, and other neurological disorders and tested this hypothesis using genetic and cognitive data from the UK Biobank (UKB) [13].

## 2 Materials and Methods

### 2.1 Cohort

The UKB is a population‐based prospective cohort study described elsewhere [13]. Participants aged 40–69 were recruited between 2006 and 2010. Data were collected through questionnaires, interviews, physical and functional assessments, imaging, and biological sampling [13]. Participants provided informed consent and can withdraw anytime, after which their data are excluded from further analyses.

### 2.2 Case–Control Definitions

MS status was determined using UKB “first occurrences” (Category 1712), which identify the first occurrence of a set of diagnostic codes mapped to three‐character ICD‐10 codes; cases were those with the code G35. To differentiate prevalent from incident cases, accounting for diagnostic and recording lag, we evaluated whether the initial diagnostic code appeared before or within 10 years of participants' baseline assessment [14]. Similarly, we identified participants with migraine (G43) as a negative disease control. We identified individuals with PD and all‐cause dementia, utilising algorithmically‐defined outcomes (Category 42), which use combinations of coded information for selected conditions.

We restricted analysis to participants of European genetic ancestry (Data‐Field 22,006) and included prevalent cases, creating five mutually‐exclusive cohorts:

- MS: Participants with an MS code ≤ 10 years after recruitment.
- PD: Participants with no code for MS and a PD code ≤ 10 years after recruitment.
- Dementia: Participants with no code for MS or PD and a dementia code ≤ 10 years after recruitment.
- Migraine: Participants with no code for MS, PD, or Dementia and a migraine code ≤ 10 years after recruitment.
- Controls: Participants with no code for MS, PD, dementia or migraine.

### 2.3 Exposure Definitions

Genotype and quality control protocols are described elsewhere [13]. Genotypes for rs10191329^A^ were obtained from UKB and extracted using the ‘‐recode AD’ flag in PLINK version 2 [15]. The imputation quality score was 0.98, indicating high‐quality imputation. Participants with missing rs10191329^A^ data were excluded. The hard call dosage for the A allele was obtained as follows:

- rs10191329^A^ allelic dosage < 0.1: non‐carriers
- rs10191329^A^ allelic dosage > 0.9 and < 1.1: heterozygous
- rs10191329^A^ allelic dosage > 1.9: homozygous

Genetic principal components (PCs) were supplied by UKB (Data‐Field 22,009).

### 2.4 Outcome Definitions

We used self‐reported disability claims (Data‐Field 6146) as a proxy of physical disability, as it reflects objective, long‐term functional limitation, and created a binary variable indicating whether participants received any form of disability allowance (Supplementary Table 1).

For our cognitive outcomes, we used tests administered at recruitment with *N* > 100,000, excluding ‘pairs matching’, which has low test–retest reliability and correlation with formal testing [16]. We assessed ‘reaction time’ (Data‐Field 20,023) in a computerised card‐matching task, with higher scores indicating slower responses. Fluid intelligence (Data‐Field 20,016) was measured as the total number of correct answers out of 13, with higher scores indicating better performance. ‘Prospective memory’ (Data‐Field 20,018) in a task requiring participants to override a prompt and select the correct target was assessed as a binary outcome with success defined as correct recall on first attempt (Supplementary Table 1). We confirmed that MS, PD, and dementia cases showed the expected pattern of poorer cognitive performance compared to controls and migraine cohorts (Supplementary Table 2, Figure S1).

### 2.5 Statistical Analysis

We first explored the relationship between rs10191329^A^ and disability claims using logistic regression adjusted for age, sex, the first four PCs, and the Townsend deprivation index. We then validated our cognitive outcomes by examining their associations with prevalent neurological disease, adjusting for age and sex.

For the primary analysis, we assessed the relationship between rs10191329^A^ and cognitive outcomes using regression stratified by disease status (separately for each cohort). We applied linear regression to reaction time and fluid intelligence, logistic regression to prospective memory, and used additive genetic models adjusted for age, sex, the first four PCs and the Townsend deprivation index. Linear outcomes (reaction time, fluid intelligence) were rank‐inverse normal transformed (RINT) to meet linear‐model assumptions. *p*‐values are quoted as two‐tailed *p*‐values testing the null hypothesis that the regression coefficient for the association between rs10191329^A^ and the outcome was equal to 0.

We performed sensitivity analyses without covariate adjustment, under dominant and recessive models. As an additional sensitivity analysis to account for the impact of education, we included the education leaving age (Data‐Field 845) as an additional covariate in the models. We applied a false discovery rate (FDR) correction to account for multiple testing, adjusting for all terms simultaneously.

We performed power calculations, using reaction time as an example due to its large sample size. We simulated a normal distribution with mean 0 and SD 1 and performed 1000 bootstrap simulations over different effect sizes, varying the number of cases. For each simulation, we used univariable linear models, regressing the genotype on reaction time. Power at the 0.05 alpha level was defined as the proportion of bootstrap iterations with two‐tailed *p*‐values of < 0.05.

### 2.6 Computing

Analysis was conducted using R version 4.4.1, and utilising Queen Mary's Apocrita HPC [17].

## 3 Results

### 3.1 Cohort Characteristics

After excluding participants of non‐European ancestry and those with missing rs10191329 genotypes (Supplementary Figure 2), we included 399,031 participants, comprising 373,530 controls (median [IQR] age 58 [51–63], 53% female), 2337 dementia (median [IQR] age 65 [62–68], 47.7% female), 19,672 migraine (median [IQR] age 56 [49–62], 75.4% female), 2026 MS (median [IQR] age 56 [49–62], 72.7% female) and 1466 PD (median [IQR] age 63 [59–66], 39.1% female) cases. The minor allele frequency of rs10191329^A^ was similar across groups. Median age at MS report was 44 (IQR 36–52), consistent with previous estimates [18]. Participant demographics are presented in Supplementary Table 3.

### 3.2 Rs10191329^A^ Weakly Correlates With Disability Claims in MS

**FIGURE 1:**
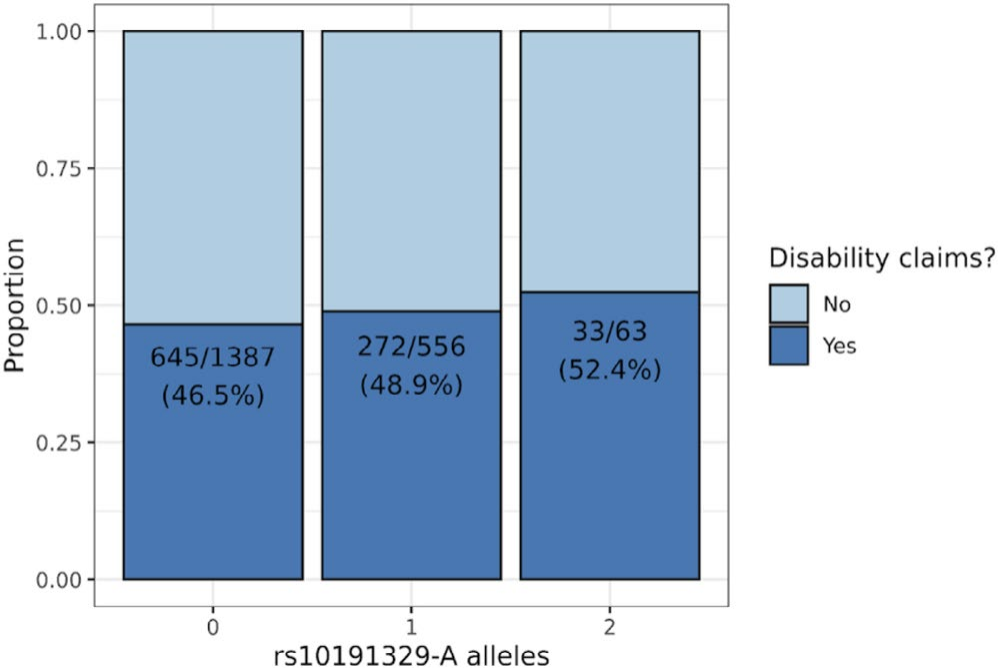
Stacked barplots showing the association between rs10191329 genotype and self‐reported physical disability claims among UK Biobank participants with MS. The counts shown reflect the number of individuals reporting any claim divided by the total number with MS within each genotype group. The dark blue bars indicate the proportion of individuals making at least one type of disability claim, and the light blue bars indicate the proportion making no claims.

We observed a weak association between rs10191329^A^ and disability claims in MS (N_MS_ = 2006). Carriers of two A alleles were 5.9% more likely to claim disability allowance than common allele homozygotes (52.4% vs. 46.5%). Regression analysis showed a directionally concordant association but did not reach statistical significance (OR 1.03, 95% CI 0.99 to 1.07, *p* = 0.15; Figure 1).

### 3.3 Rs10191329^A^ Correlates With Impaired Cognition

**FIGURE 2:**
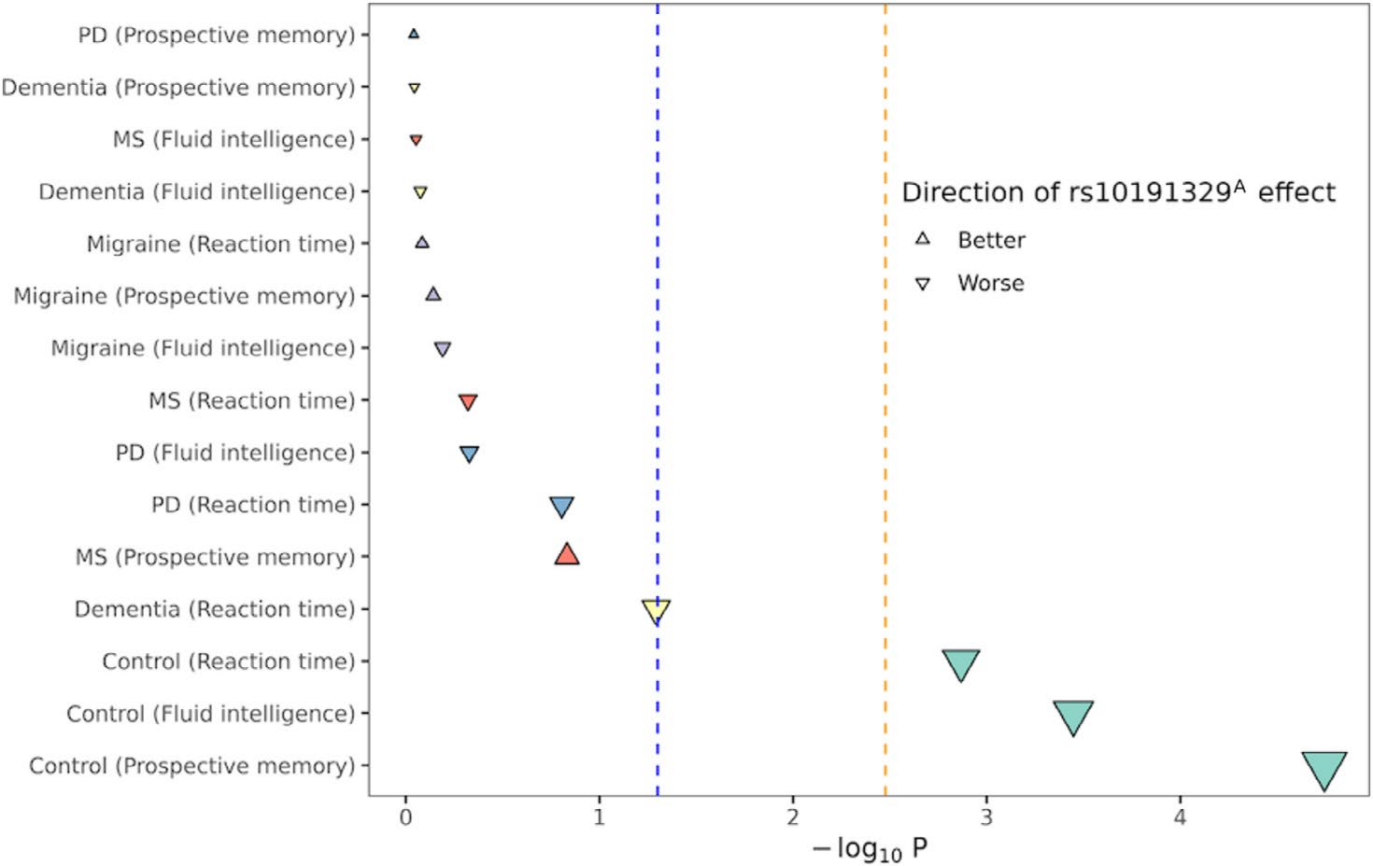
Plot showing the effect directions and −log10(P) values from regression models assessing the relationship between rs10191329 genotype and cognitive outcomes in each disease group. Each point represents a regression coefficient. The shape of the point indicates the effect direction, with downward triangles indicating that rs10191329^A^ was associated with worse cognition, and vice versa. The colour of the dots indicates the disease group, and the y‐axis indicates the specific coefficient (i.e., combination of disease group and test). Points are ordered by their strength of association such that the strongest signals (i.e., largest −log10(P) values) are at the bottom of the plot. The x‐axis shows the strength of association, as does the size of the point. The dotted blue and orange vertical lines indicate p = 0.05 and P_Bonferroni_ = 0.05, respectively. Model coefficients are adjusted for age, sex, Townsend deprivation index, and the first four genetic principal components.

Rs10191329^A^ was associated with slower reaction time (𝜷 = 0.01 SD per allele, 95% CI 0.004 to 0.015, *p* = 0.001), lower fluid intelligence (𝜷 = −0.02 SD per allele, 95% CI −0.03 to −0.01, *p* = 0.0004), and higher error rates on the prospective memory task (OR 1.06, 95% CI 1.03 to 1.09, *p* = 0.00002) in controls (Figure 2). No other associations reached study‐wide significance (FDR < 5%). However, we observed directionally concordant associations between rs10191329^A^ and slower reaction time in MS, PD, and dementia, with lower fluid intelligence in all cohorts, and impaired prospective memory in dementia. Results were consistent in sensitivity analyses adjusting for education and considering alternative genetic models (Supplementary Table 4).

### 3.4 Power Calculations

Power calculations indicated we were able to detect a per‐allele increase in reaction time of > 0.15‐SD (98% power) in MS (*n* = 1,993), but not weaker associations (e.g., 63% power for a 0.1‐SD per‐allele increase). We therefore had high power to detect moderate per‐allele effects (> 0.15 SD) but limited power for the smaller effects observed in the control cohort (≤ 0.1 SD).

## 4 Discussion

Rs10191329^A^–a modifier of physical disability in MS–is associated with impaired cognitive outcomes in ~370,000 healthy adults. Despite limited power to detect analogous effects in MS or other neurological disorders, analyses suggested directionally concordant associations across health and disease.

Prior evidence has implicated rs10191329^A^ in MS progression, correlating the minor (A) allele with greater physical disability, increased radiological and neuropathological disease burden, and biomarker (neurofilament light chain) evidence of increased neurodegeneration in MS [2, 3, 4, 19]. Our results show that rs10191329^A^ is weakly associated with impaired cognitive performance, suggesting that its effects may extend beyond MS through shared mechanisms [2, 4]. One possible pathway is education‐mediated cognitive reserve, as genetically‐predicted educational attainment is associated with a milder MS disease course [2]; however, as adjustment for education leaving age–a proxy of educational attainment–did not significantly attenuate these findings, it is unlikely this association is explained by educational attainment alone.

Reliance on cross‐sectional, non–MS‐specific outcomes likely attenuates detectable associations and contributes to the small effect sizes and the small proportion of variance explained. Importantly, none of the outcomes used are specifically validated as outcome measures for Multiple Sclerosis, but rather were included in the UK Biobank as pragmatic outcomes to explore variation across a range of health and disease. In addition, prevalent UKB cases are biased toward people with milder disease, which may further reduce sensitivity to detect genetic effects on disease progression. In addition, our study was restricted to a limited cognitive test set, hampered by limited power due to sample size for examining within‐disease associations, we did not have access to a comparable replication dataset and we did not test our findings in people of non‐European ancestry.

In summary, using population‐scale cognitive testing of > 370,000 adults, we identify weak but directionally consistent associations between rs10191329^A^ and lower cognitive outcomes in healthy adults, which are compatible with the hypothesis that this variant might influence MS severity through effects on brain health. Further work in large disease‐specific cohorts is required to explore the impact of rs10191329^A^ on cognition in MS, other neurological disorders, and in health.

## Author Contributions

All authors contributed to the manuscript. I.Z., B.M.J. and R.D. contributed to the conception and design of the study. I.Z., S.W., B.M.J. and R.D. contributed to the acquisition and analysis of data. I.Z., B.M.J. and R.D. contributed to drafting the text or preparing the figures. All authors contributed to revising the manuscript for submission.

## Funding

IZ was supported by a National Institute of Health Research (NIHR) Academic Foundation Programme post. PS was supported by the European Union's Horizon 2020 Research and Innovation program (Grant MultipleMS, EU RIA 733161) and a grant from the Margaretha av. Ugglas Foundation. BMJ was supported by a Guarantors of Brain post‐doctoral fellowship at the outset of this project, and is currently funded by an NIHR Academic Clinical Lectureship.

## Conflicts of Interest

The authors declare no conflicts of interest.

## Supporting information


**Figure S1:** A—Boxplots displaying the reaction time (in ms) for UK Biobank participants in each of the mutually‐excluded disease cohorts (x‐axis). For clarity, the y‐axis shows reaction times on the log10 scale. Higher reaction times indicate poorer performance. B—as per A, but showing the raw fluid intelligence scores for each cohort. Scores are shown out of 13, with higher scores indicating better performance. C—stacked barplots showing the performance of each disease cohort on a prospective memory task. The outcomes shown are incorrect (the worst outcome), correct on the 1st attempt (best outcome), and correct on the 2nd attempt. A higher proportion of respondents correct at the 1st attempt indicates better performance.
**Figure S2:** Flow diagram indicating the numbers of participants at each stage of the inclusion and exclusion criteria. In total, the final analysis cohort comprised 399,031 White British UKB participants.
**Table S1:** Description of outcome measures used.
**Table S2:** Associations between prevalent neurological diseases and cognitive test scores. The table shows the output of multivariable linear regression (for reaction time and fluid intelligence) or logistic regression (for prospective memory) models adjusted for age and sex. The beta coefficients displayed reflect the association between the stated disease cohort and the cognitive test score, with healthy controls as the reference category. For each outcome, the direction of effect is simplified as ‘better’ or ‘worse’ than controls. For reaction time and fluid intelligence, the outcome was subjected to rank inverse normalisation prior to model fitting, and so the beta coefficient is on this scale. For prospective memory, an incorrect response was coded as a ‘1’ and a correct response as a ‘0’, and so the beta coefficient refers to the log(Odds Ratio) of an incorrect response, i.e., beta > 0 ~ worse performance. False Discovery Rate (FDR) adjusted *p*‐values reflect a global FDR, accounting for a total of 21 terms (intercept, age, sex, and 4 disease terms). FDR values with < 0.05 are indicated with a *.
**Table S3:** Demographics and raw cognitive test scores of included participants, stratified by disease cohort. Quantitative variables are presented as median and interquartile range, and categorical variables are presented as n and %.
**Table S4:** The table shows the association between the rs10191329 genotype and cognitive test performance in each disease cohort. Models were adjusted for age, sex, the Townsend deprivation index and the first four genetic principal components. The ‘genotype N' column shows the number within each disease cohort for the stated test with each of the three genotypes (CC, CA, or AA). Sensitivity analyses were performed, including an unadjusted model, use of a dominant genetic model, and use of a recessive model, as well as use of educational leaving age. For linear models (reaction time, fluid intelligence), estimates represent regression beta coefficients, whereas for logistic regression models (prospective memory), estimates represent log odds ratios.

## Data Availability

All code for analysis is provided on: https://github.com/izimianiti/UKB‐MS‐severity‐allele‐cognition. UK Biobank data are available on request from https://www.ukbiobank.ac.uk/. This research was conducted under approved application 78,867.
